# Association between Temporomandibular Joint Disorder and Weight Changes: A Longitudinal Follow-Up Study Using a National Health Screening Cohort

**DOI:** 10.3390/ijerph182211793

**Published:** 2021-11-10

**Authors:** So Young Kim, Dae Myoung Yoo, Soo-Hwan Byun, Chanyang Min, Ji Hee Kim, Mi Jung Kwon, Joo-Hee Kim, Hyo Geun Choi

**Affiliations:** 1 Department of Otorhinolaryngology-Head & Neck Surgery, CHA Bundang Medical Center, CHA University, Seongnam 13496, Korea; sossi81@hanmail.net; 2 Hallym Data Science Laboratory, Hallym University College of Medicine, Anyang 14068, Korea; ydm1285@naver.com (D.M.Y.); joicemin@naver.com (C.M.); 3 Department of Oral & Maxillofacial Surgery, Dentistry, Hallym University College of Medicine, Anyang 14068, Korea; purheit@daum.net; 4 Graduate School of Public Health, Seoul National University, Seoul 08826, Korea; 5 Department of Neurosurgery, Hallym University College of Medicine, Anyang 14068, Korea; kimjihee.ns@gmail.com; 6 Department of Pathology, Hallym University College of Medicine, Anyang 14068, Korea; mulank@hanmail.net; 7 Division of Pulmonary, Allergy, and Critical Care Medicine, Department of Medicine, Hallym University College of Medicine, Anyang 14068, Korea; luxjhee@gmail.com; 8 Department of Otorhinolaryngology-Head & Neck Surgery, Hallym University College of Medicine, Anyang 14068, Korea

**Keywords:** temporomandibular joint disorder, obesity, risk factors, cohort studies

## Abstract

This study aimed to investigate BMI changes following a temporomandibular joint disorder (TMJD) diagnosis. The Korean National Health Insurance Service-Health Screening Cohort from 2002 to 2015 was used. In Study I, 1808 patients with TMJD (TMJD I) were matched with 7232 participants in comparison group I. The change in BMI was compared between the TMJD I and comparison I groups for 1 year. In study II, 1621 patients with TMJD (TMJD II) were matched with 6484 participants in comparison group II participants. The change in BMI was compared between the TMJD II and comparison II groups for 2 years. In Study I, the BMI change was not associated with TMJD. In Study II, the BMI change was associated with TMJD in the interaction of the linear mixed model (*p* = 0.003). The estimated value (EV) of the linear mixed model was −0.082. The interaction was significant in women < 60 years old, women ≥ 60 years old, and the obese I category. TMJD was not associated with BMI changes after 1–2 years in the overall population. In women and obese patients, TMJD was associated with a decrease in BMI after 2 years.

## 1. Introduction

Temporomandibular disorder (TMD) is a group of disorders that includes temporomandibular joint (TMJ) pain and dysfunction. It might originate from changes in the structure and function of the TMJ, masticator muscle, and osseous structure [1]. It is the most common orofacial pain, and its prevalence is ~20% of the general population [2]. The peak onset age of TMD was reported to be between 20 and 40 years old and to mainly present in women [3]. In Korea, 11.8% of the general population experiences TMD [4]. The risk factors for TMD have been reported to be obesity, occlusion abnormalities, bruxism, trauma, osteoporosis, stress, anxiety, and depression [5,6]. Temporomandibular joint disorder (TMJD) is one of the common etiologies of TMD, and is prevalent in older population without gender preference [7]. 

The common symptoms of TMD are pain and difficulty during mastication [1]. It results in problems in the oral preparatory phase with solid (33%) and liquid (28%) swallowing [8]. TMD can cause headaches, neck pain, body pain, and dietary problems [9,10]. It has also been reported that TMD can cause weight loss (26%) [8]. However, this last study was not compared with an appropriate comparison group and studied in a limited population (*n* = 178) using only a self-report survey [8].

We hypothesized that TMJD might be associated with weight loss, as it is closely associated to mastication function. However, this relationship had not previously been evaluated using rigorous methods. We evaluated this association using health check-up data that were objectively measured and compared the data with the matched comparison participants using a large population-based cohort.

## 2. Materials and Methods

### 2.1. Study Population

This study was approved by the Ethics Committee of Hallym University (2019-10-023). The Institutional Review Board waived the requirement of written informed consent. This study used the Korean National Health Insurance Service–Health Screening Cohort data [11].

### 2.2. Definition of Temporomandibular Joint Disorder (Independent Variable)

The participants had been diagnosed under the diagnostic code for TMJD (ICD-10: K07.6 (temporomandibular joint disorders)). Participants who had histories of two or more clinical visits presenting with TMJD were included [9,12]. 

### 2.3. Definition of Weight Change (Dependent Variable)

In study I, BMI change safter one year from the TMJD diagnosis were followed up. In study II, BMI changes after two years from the diagnosis of TMJD were followed up (Study II).

### 2.4. Participant Selection

From total cohort data from 2002–2015 with 514,866 participants, 4627 TMJD participants were enrolled. TMJD participants who did not provide follow-up data (*n* = 1480) were excluded. From the identical total cohort data, comparison participants who had no history of TMJD were selected (*n* = 510,239). Comparison participants who had a history of TMJD were excluded (*n* = 6659). The comparison participants were randomly selected to prevent selection bias. The 1917 comparison participants provided 1-year follow-up data. The 1722 comparison participants provided 2-year follow-up data. A total of 492 comparison participants provided both one-year and two-year follow-up data.

**Figure 1:**
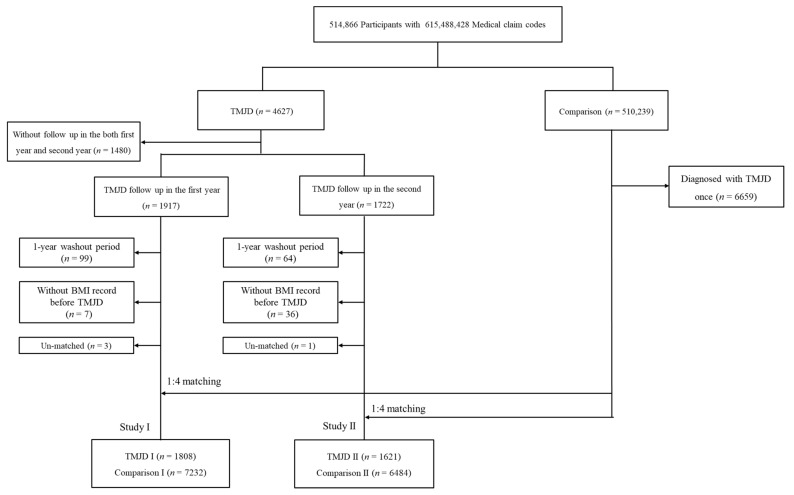
A schematic illustration of the participant selection process that was used in the present study. Of a total of 514,866 participants, 4627 TMJD participants were selected. Among them, we excluded participants without histories of first- or second-year follow-up records (*n* = 1480). Then, participants were categorized as TMJD I with a first year of follow-up (*n* = 1917) and TMJD II with a second year of follow-up (1722). A total of 492 TMJD participants were included in both groups. After the exclusion of 1 year of wash out, participants without BMI records, and unmatched participants, TMJD participants were 1:4 matched with comparison participants.

In Study I, 99 TMJD participants were excluded due to a diagnosed history of TMJD before 2002 (washout periods). TMJD participants who did not have BMI records were excluded (*n* = 7). TMJD participants were 1:4 matched with comparison participants for age, sex, income, region of residence, and obesity. The index date of each TMJD participant was defined as the time of diagnosis of TMJD. The index date of the comparison participants was matched with their matched TMJD participants. Three TMJD participants and 496,348 comparison participants were excluded due to unmatched data. Finally, 1808 participants in the TMJD I group and 7232 participants in the comparison I group were selected (Figure 1).

In Study II, TMJD participants who had been followed up for 2 or more years were selected. The TMD participants and their matched comparison II participants were enrolled with identical inclusion and exclusion criteria. There were 64 patients who were diagnosed with TMJD before 2002 and 36 patients who did not have BMI records. These TMJD patients were excluded from the TMJD II group. Finally, 1621 TMJD II participants and 6484 comparison II participants were enrolled (Figure 1).

### 2.5. Covariates

The 40 years and older study population was divided into 10 age groups with 5-year intervals. Level of income was divided into 5 classes [13]. Regions of residence was divided into urban and rural areas [11]. Participants’ histories of tobacco smoking and alcohol consumption were surveyed. Participants’ systolic blood pressure, diastolic blood pressure, fasting blood glucose, and total cholesterol levels were measured [11]. The Charlson Comorbidity Index (CCI) was calculated as a continuous variable (between 0 (no comorbidities) and 29 (multiple comorbidities)). BMI (kg/m^2^) was classified as underweight (<18.5), normal (≥18.5 to <23), overweight (≥23 to <25), obese I (≥25 to <30), or obese II (≥30) [14].

### 2.6. Statistical Analyses

The chi-square test was used to calculate the differences in the rates of general characteristics.

Paired *t*-tests were used to analyze the differences in weight pre- and post-TMJD diagnosis. A linear mixed model was used to analyze the interaction and estimated value (EV). The independent variables of age, sex, income, region of residence, TMJD, and time of measurement were used as the fixed effects. BMI, systolic blood pressure, diastolic blood pressure, fasting blood glucose, total cholesterol level, smoking status, alcohol consumption, and CCI scores were used as random effects. A first-order autoregressive model was selected as the repeated covariance type, which considered the correlation of each participant’s iteration. The statistical analysis model of the linear mixed model is as follows.

$$Y_{i}=X_{i1}\beta_{1\;}+..\;.+X_{ip}\beta_{p}+Z_{i1}u_{i}+..\;.+Z_{iq}u_{q}+e_{i},\;\mathrm{for}\;\mathrm{all}\;i=1,\ldots ,n\;$$

where Y= ($Y_{1},\ldots ,\;Y_{n}$)′, X is the n×p matrix of covariates with fixed effects $\beta ={\left(\beta_{1},\ldots ,\;\beta_{p}\right)}^{\prime }$, Z is the n×q matrix of covariates with random effects, $u={\left(u_{1},\ldots ,\;u_{q}\right)}^{\prime }\sim \;N\left(0,\tau I_{q}\right)$, and the residual error vector $e={\left(e_{1},\ldots ,\;e_{n}\right)}^{\prime }\sim \;N\left(0,\tau I_{n}\right),$.

Subgroup analyses were conducted according to age and sex (<60 years and ≥60 years; men and women) and by obesity status (underweight, normal, overweight, obese I, obese II).

Two-tailed analyses were conducted. Statistical significance was defined as *p* < 0.05/2 to avoid type I error caused by the comparison of two studies. SAS version 9.4 (SAS Institute Inc., Cary, NC, USA) was used.

## 3. Results

The general characteristics of age, sex, income, and region of residence were exactly the same between the TMJD and comparison groups in both Study I and Study II due to matching (Table 1).

The paired *t*-test did not show differences between the pre- and post-TMJD 1-year records of the participants in the TMJD I and comparison I groups (Table 2). The interaction in the linear mixed model did not reach statistical significance in Study I. The decrease in BMI was significant in the TMJD I group of men aged <60 years, but this change was not significant in the interaction model.

In the subgroup analyses according to obesity status, the change in weight was significant in both the TMJD I and comparison I groups, except for the overweight group (Table 3). In the underweight/normal weight category, the BMIs of the participants in both the TMJD I and comparison I groups increased. In the obese I category, the BMIs of the participants in the TMJD I group decreased and those in participants in comparison I group increased. In the obese II category, the BMIs of the participants in both the TMJD I and comparison I groups decreased. However, none of these changes were statistically significant in the interaction model.

The paired *t*-test did not show differences in the pre- and post-TMJD 2-year records among all participants in the TMJD II and comparison II groups (Table 4). On the other hand, the interaction in the linear mixed model reached statistical significance (*p* = 0.003), and the EV of the linear mixed model was −0.082. A decrease in BMI was found in the TMJD II group in women ≥ 60 years old, while an increase in BMI was observed in the comparison II group in men < 60 years old and women < 60 years old. The interaction was significant in women < 60 years old and women ≥ 60 years old. The EV was −0.109 for women < 60 years old and −0.272 in women ≥ 60 years old.

In the subgroup analyses according to obesity status, an increase in BMI was observed in underweight/normal weight individuals in the TMJD II and comparison II groups (Table 5). A decrease in BMI was found in obese individuals in the TMJD II and comparison II groups. The interaction model was significant in the obese I category, and its EV was −0.200.

## 4. Discussion

It was found that the change in BMI was significant only in Study II, which measured the 2-year change in patients with TMJD in the present study. A decrease in BMI was observed in the TMJD II group compared with the comparison II group only in women and the obese I category. This association was not found in any of the subgroups of Study I, which had a 1-year follow-up. This is the first study that reports the change in BMI in TMJD participants compared to matched comparison participants.

We believe this change in BMI is clinically meaningful, even though statistical significance was observed only in the women and obese I subgroups with the 2-year follow-up. The BMI change over 1 or 2 years was not significant in most of the subgroups, and the change in BMI was very small. As this study enrolled a large number of participants, statistical significance was detected with these minimal changes in BMI. In this study, among the statistically significant values, the largest EV was −0.272. This means that the BMIs of the participants in the TMJD group decreased by −0.272 compared to those of participants in of the comparison group. If the height of a participant were 170 cm, their BMI would change by −0.78 kg in 2 years.

The association of TMD with BMI has been suggested in several prior studies with differing results [5,15,16,17,18]. In a cross-sectional study, TMD was associated with low BMI in women (adjusted odds ratio (aOR) = 1.44, 95% confidence interval (95% CI) = 1.09–1.93, *p* = 0.037) [5]. However, other cross-sectional studies demonstrated no association between BMI and TMD in adolescents [15] or the general population [16]. On the other hand, overweight (BMI ≥ 25) was associated with frequent pain-associated TMD symptoms among Finnish conscripts (aOR = 1.23, 95% CI = 1.01–1.49) [18]. Another cross-sectional study in an adult population suggested an association of TMD with obesity in a univariate analysis. These differing observed associations between TMD and weight loss may originate from the limited numbers of participants in the above studies. Previously, few studies reported such an association compared with comparison groups. In addition, the potential effects of the aging process on weight loss could not be excluded in previous studies, because they did not have comparison participants who matched for age and BMI. As follow-up durations were not defined in most prior studies, the temporal association between TMD and weight loss could not be estimated.

TMJD could be associated with decreased BMI due to changes in eating behaviors and stress factors associated with TMJD. Patients with TMD may have weakened biting force, which impairs masticatory movement [19]. The pain of TMJ was reported to affect dietary intake, which leads to the avoidance of specific foods, such as meat and apples [20]. In addition, patients with TMD showed a higher rate of mental stress [5]. Mental stress was reported to delay the gastrointestinal transit time and peak glucose response, which decreased the appetite and dietary intake [21]. Mastication difficulties, pain, and psychological stress could decrease a person’s dietary intake, which may result in a decreased BMI in patients with TMD. Because obesity was suggested to be one of the factors associated with TMD, the present study analyzed the impact of TMD on BMI changes according to different BMI groups. As a result, the obese I population showed an association of TMD with decreased BMI. Compared with male subgroups, female subgroups demonstrated decreased BMI associated with TMJD in this study. Similar to the present result, a previous study also reported a sex-specific association of TMD with decreased BMI [5]. They supposed that a higher susceptibility to psychological stress and anxiety associated with TMD in women may be linked with a decreased BMI [5].

In the overall population and other subgroups, TMJD was not associated with BMI changes in the present study. Several explanations could support the present results. First, the decreased dietary intake could be compensated by the substitution of foods by patients with TMJD. A survey described the modifications of diet in patients with TMD [22]. Approximately 77.6% (66/85) of TMD patients modified their diet, including by cutting food into smaller pieces (71.8% [61/85]), softening (42.4% [36/85]), and mashing (40% [34/85]) their food [22]. By these efforts, the potential risk of nutritional deficits could be prevented in patients with TMJD. Second, a decrease in BMI could relieve the pain and other symptoms of TMJD, which alleviates dietary disturbances in TMJD patients. Third, the contribution of TMJD to BMI changes was not considerable, and many other factors could mediate the link between TMJD and BMI changes. For instance, a previous study reported that the association of TMD with obesity was not evident after adjusting for sex and other comorbidities, such as headaches and obstructive sleep apnea [17]. As the present study comprehensively adjusted for potential confounders, that could explain why the association of TMJD with BMI changes was not statistically significant.

The present study used a large nationwide cohort population. Many comparison participants were matched for age, sex, income, region of residence, and obesity status, and were randomly selected. The variables were reliable and were collected from national health insurance and health check-up data. Participants’ past medical histories were based on diagnostic codes, and laboratory measures were used for the levels of blood pressure, blood glucose, and total cholesterol. The accuracy of BMI data could be guaranteed by objective measures during health check-ups. In addition, the duration of follow-up was classified into 1 year and 2 years, so that we could assess the sensitivity of the association of TMJD with weight loss. To minimize the bias due to multiple comparisons, the Bonferroni correction was conducted. However, the long-term effects of TMJD on weight loss could not be evaluated in the present cohort data. To compensate for the short follow-up duration of this study, two independent studies were conducted with 1-year and 2-year follow-up durations. This study was based on health check-up data, thus selection bias cannot be excluded. Some information about our cohort was not accessible, which could induce information bias. The severity of TMJD was heterogeneous, and the treatment histories of patients with TMJD were not available in this study. Because this study was based on diagnostic code ICD10, the etiology of TMJD could not be isolated. TMJD could be mixed with TMD. However, TMD is more common in children and adolescents [23]. On the other hand, TMJD was more common in the elderly population in our study and was associated with TMJ degeneration [7]. Although many comorbidities, anthropometric data, and lifestyle factors were adjusted, confounders, such as nutritional factors, may have remained. Last, a detailed etiology for weight loss could not be isolated in the present data.

## 5. Conclusions

Patients with TMJD did not show more changes in BMI than comparison participants in the overall population. A decrease in BMI associated with TMJD was observed in a subpopulation of women and obese patients with a 2-year follow-up duration.

## Figures and Tables

**Table 1:** General characteristics of participants.

Characteristics			Study I					Study II		
	Total (*n*)	TMJD I (*n*, %)	Total (*n*)	Comparison I (*n*, %)	*p*-Value	Total (*n*)	TMJD II (*n*, %)	Total (*n*)	Comparison II (*n*, %)	*p*-Value
Age (years)					1.000					1.000
40–44	1808	66 (3.7)	7232	264 (3.7)		1621	64 (4.0)	6484	256 (4.0)	
45–49	1808	239 (13.2)	7232	956 (13.2)		1621	199 (12.3)	6484	796 (12.3)	
50–54	1808	326 (18.0)	7232	1304 (18.0)		1621	358 (22.1)	6484	1432 (22.1)	
55–59	1808	359 (19.9)	7232	1436 (19.9)		1621	265 (16.4)	6484	1060 (16.4)	
60–64	1808	228 (12.6)	7232	912 (12.6)		1621	192 (11.8)	6484	768 (11.8)	
65–69	1808	247 (13.7)	7232	988 (13.7)		1621	209 (12.9)	6484	836 (12.9)	
70–74	1808	188 (10.4)	7232	752 (10.4)		1621	221 (13.6)	6484	884 (13.6)	
75–79	1808	131 (7.3)	7232	524 (7.3)		1621	80 (4.9)	6484	320 (4.9)	
80–84	1808	21 (1.2)	7232	84 (1.2)		1621	30 (1.9)	6484	120 (1.9)	
85+	1808	3 (0.2)	7232	12 (0.2)		1621	3 (0.2)	6484	12 (0.2)	
Sex					1.000					1.000
Male	1808	849 (47.0)	7232	3396 (47.0)		1621	737 (45.5)	6484	2948 (45.5)	
Female	1808	959 (53.0)	7232	3836 (53.0)		1621	884 (54.5)	6484	3536 (54.5)	
Income					1.000					1.000
1 (lowest)	1808	283 (15.7)	7232	1132 (15.7)		1621	244 (15.1)	6484	976 (15.1)	
2	1808	252 (13.9)	7232	1008 (13.9)		1621	231 (14.3)	6484	924 (14.3)	
3	1808	281 (15.5)	7232	1124 (15.5)		1621	277 (17.1)	6484	1108 (17.1)	
4	1808	375 (20.7)	7232	1500 (20.7)		1621	340 (21.0)	6484	1360 (21.0)	
5 (highest)	1808	617 (34.1)	7232	2468 (34.1)		1621	529 (32.6)	6484	2116 (32.6)	
Region of residence					1.000					1.000
Urban	1808	740 (40.9)	7232	2960 (40.9)		1621	675 (41.6)	6484	2700 (41.6)	
Rural	1808	1068 (59.1)	7232	4272 (59.1)		1621	946 (58.4)	6484	3784 (58.4)	
Obesity †					1.000					1.000
Underweight	1808	43 (2.4)	7232	172 (2.4)		1621	38 (2.3)	6484	152 (2.3)	
Normal	1808	725 (40.1)	7232	2900 (40.1)		1621	637 (39.3)	6484	2548 (39.3)	
Overweight	1808	529 (29.3)	7232	2116 (29.3)		1621	472 (29.1)	6484	1888 (29.1)	
Obese I	1808	474 (26.2)	7232	1896 (26.2)		1621	445 (27.5)	6484	1780 (27.5)	
Obese II	1808	37 (2.1)	7232	148 (2.1)		1621	29 (1.8)	6484	116 (1.8)	
Smoking status					0.036 *					0.002 *
Nonsmoker	1808	1346 (74.5)	7232	5357 (74.1)		1621	1250 (77.1)	6484	4820 (74.3)	
Past smoker	1808	228 (12.6)	7232	801 (11.1)		1621	178 (11.0)	6484	671 (10.4)	
Current smoker	1808	234 (12.9)	7232	1074 (14.9)		1621	193 (11.9)	6484	993 (15.3)	
Alcohol consumption					0.572					0.879
<1 time a week	1808	1319 (73.0)	7232	5228 (72.3)		1621	1197 (73.8)	6484	4800 (74.0)	
≥1 time a week	1808	489 (27.1)	7232	2004 (27.7)		1621	424 (26.2)	6484	1684 (26.0)	
Systolic blood pressure					0.002 *					0.007 *
<120 mmHg	1808	629 (34.8)	7232	2338 (32.3)		1621	561 (34.6)	6484	2078 (32.1)	
120–139 mmHg	1808	900 (49.8)	7232	3531 (48.8)		1621	787 (48.6)	6484	3100 (47.8)	
≥140 mmHg	1808	279 (15.4)	7232	1363 (18.9)		1621	273 (16.8)	6484	1306 (20.1)	
Diastolic blood pressure					0.002 *					0.005 *
<80 mmHg	1808	930 (51.4)	7232	3524 (48.7)		1621	839 (51.8)	6484	3079 (47.5)	
80–89 mmHg	1808	647 (35.8)	7232	2545 (35.2)		1621	550 (33.9)	6484	2331 (36.0)	
≥90 mmHg	1808	231 (12.8)	7232	1163 (16.1)		1621	232 (14.3)	6484	1074 (16.6)	
Fasting blood glucose					0.188					0.381
<100 mg/dL	1808	1201 (66.4)	7232	4746 (65.6)		1621	1104 (68.1)	6484	4359 (67.2)	
100–125 mg/dL	1808	497 (27.5)	7232	1957 (27.1)		1621	411 (25.4)	6484	1636 (25.2)	
≥126 mg/dL	1808	110 (6.1)	7232	529 (7.3)		1621	106 (6.5)	6484	489 (7.5)	
Total cholesterol					0.016 *					0.510
<200 mg/dL	1808	999 (55.3)	7232	3788 (52.4)		1621	870 (53.7)	6484	3377 (52.1)	
200–239 mg/dL	1808	607 (33.6)	7232	2470 (34.2)		1621	544 (33.6)	6484	2261 (34.9)	
≥240 mg/dL	1808	202 (11.2)	7232	974 (13.5)		1621	207 (12.8)	6484	846 (13.1)	
CCI score					0.562					0.297
0	1808	1286 (71.1)	7232	5221 (72.2)		1621	1162 (71.7)	6484	4737 (73.1)	
1	1808	259 (14.3)	7232	974 (13.5)		1621	225 (13.9)	6484	867 (13.4)	
2	1808	143 (7.9)	7232	526 (7.3)		1621	131 (8.1)	6484	434 (6.7)	
3	1808	52 (2.9)	7232	245 (3.4)		1621	50 (3.1)	6484	205 (3.2)	
≥4	1808	68 (3.8)	7232	266 (3.7)		1621	53 (3.3)	6484	241 (3.7)	

Abbreviations: CCI, Charlson comorbidity index; TMJD, Temporomandibular joint disorder. * Chi-square test; significance was defined as *p* < 0.05. † Obesity (BMI, body mass index, kg/m^2^) was categorized as underweight (<18.5), normal (≥18.5 to <23), overweight (≥23 to <25), obese I (≥25 to <30), and obese II (≥30).

**Table 2:** Differences in mean BMI between pre- and 1-year-post-study of TMJD in Study I according to age and sex.

Characteristics	TMJD I			Comparison I			Interaction ‡	Linear Mixed Model ¶	
	Previous (Mean, SD)	Post 1yr (Mean, SD)	*p*-Value	Previous (Mean, SD)	Post 1yr (Mean, SD)	*p*-Value	*p*-Value	EV §	*p*-Value
Total participants (*n* = 9040)	23.58 ± 2.83	23.58 ± 2.83	0.957	23.62 ± 2.84	23.62 ± 2.89	0.979	0.769	−0.014	0.850
Age 40–60 years old, men (*n* = 2365)	23.92 ± 2.54	24.04 ± 2.54	0.010 *	23.98 ± 2.66	24.02 ± 2.67	0.154	0.146	0.044	0.736
Age 40–60 years old, women (*n* = 2585)	23.45 ± 3.09	23.49 ± 3.09	0.374	23.44 ± 2.92	23.47 ± 2.95	0.394	0.799	0.055	0.698
Age ≥60 years old, men (*n* = 1880)	23.23 ± 2.76	23.06 ± 2.62	0.032	23.28 ± 2.78	23.22 ± 2.83	0.092	0.208	−0.135	0.389
Age ≥60 years old, women (*n* = 2210)	23.67 ± 2.84	23.61 ± 2.91	0.397	23.73 ± 2.94	23.72 ± 3.02	0.668	0.468	−0.069	0.656

Abbreviations: CCI, Charlson Comorbidity Index; EV, estimated value; TMJD, temporomandibular joint disorders. * Paired *t*-test; significance was defined as *p* < 0.05/2. ‡ Interaction effects between time and group. § Estimated value of the linear mixed model for TMJD I group based on the comparison I group. ¶ Fixed effects were age, sex, income, region of residence, TMJD, and time of measurement. Random effects were systolic blood pressure, diastolic blood pressure, fasting blood glucose, total cholesterol, smoking, alcohol consumption, and CCI score.

**Table 3:** Differences in mean BMI between pre- and 1-year-post-study of TMJD in TMJD I and the comparison I group according to obesity.

Characteristics	TMJD I			Comparison I			Interaction ‡	Linear Mixed Model ¶	
	Previous (Mean, SD)	Post 1 Year (Mean, SD)	*p*-Value	Previous (Mean, SD)	Post 1 Year (Mean, SD)	*p*-Value	*p*-Value	EV §	*p*-Value
Underweight (*n* = 215)	17.55 ± 0.80	17.99 ± 1.09	0.008 *	17.57 ± 0.79	18.05 ± 1.46	<0.001 *	0.810	−0.046	0.807
Normal (*n* = 3625)	21.26 ± 1.16	21.49 ± 1.55	<0.001 *	21.26 ± 1.17	21.46 ± 1.63	<0.001 *	0.649	0.042	0.465
Overweight (*n* = 2645)	23.96 ± 0.56	23.91 ± 1.19	0.254	24.01 ± 0.57	23.98 ± 1.29	0.334	0.584	−0.068	0.154
Obese I (*n* = 2370)	26.58 ± 1.19	26.34 ± 1.82	<0.001 *	26.73 ± 1.26	26.47 ± 1.74	<0.001 *	0.785	−0.108	0.165
Obese II (*n* = 185)	31.99 ± 2.45	30.72 ± 3.04	0.007 *	31.52 ± 1.54	30.75 ± 2.34	<0.001 *	0.165	−0.087	0.821

Abbreviations: CCI, Charlson Comorbidity Index; EV, estimated value; TMJD, temporomandibular joint disorders. * Paired *t*-test; significance was defined as *p* < 0.05/2. ‡ Interaction effects between time and group. § Estimated value of the linear mixed model for the TMJD I group based on the comparison I group. ¶ Fixed effects were age, sex, income, region of residence, TMJD, and time of measurement. Random effects were systolic blood pressure, diastolic blood pressure, fasting blood glucose, total cholesterol, smoking, alcohol consumption, and CCI score.

**Table 4:** Differences in mean BMI between pre- and 2-year-post-study of TMJD in Study II according to age and sex.

Characteristics	TMJD II			Comparison II			Interaction ‡	Linear Mixed Model ¶	
	Previous (Mean, SD)	Post 2 Years (Mean, SD)	*p*-Value	Previous (Mean, SD)	Post 2 Years (Mean, SD)	*p*-Value	*p*-Value	EV §	*p*-Value
Total participants (*n* = 8105)	23.70 ± 2.96	23.62 ± 2.84	0.064	23.69 ± 2.82	23.72 ± 2.90	0.148	0.003 †	−0.082	0.294
Age 40–60 years, men (*n* = 2090)	24.13 ± 2.68	24.21 ± 2.55	0.156	24.07 ± 2.65	24.14 ± 2.70	0.023 *	0.879	0.047	0.740
Age 40–60 years, women (*n* = 2340)	23.38 ± 3.41	23.28 ± 2.98	0.343	23.28 ± 2.84	23.42 ± 2.87	<0.001 *	0.003 †	−0.109	0.458
Age ≥60 years, men (*n* = 1595)	23.36 ± 2.58	23.33 ± 2.83	0.763	23.39 ± 2.62	23.33 ± 2.77	0.110	0.963	0.023	0.888
Age ≥60 years, women (*n* = 2080)	23.90 ± 2.87	23.64 ± 2.88	0.001 *	24.00 ± 3.02	23.93 ± 3.14	0.109	0.023†	−0.272	0.098

Abbreviations: CCI, Charlson Comorbidity Index; EV, estimated value; TMJD, temporomandibular joint disorders. * Paired *t*-test; significance was defined as *p* < 0.05/2. † Linear mixed model; significance was defined as *p* < 0.05/2. ‡ Interaction effects between time and group. § Estimated value of the linear mixed model for the TMJD II group based on the comparison II group. ¶ Fixed effects were age, sex, income, region of residence, TMJD, and time of measurement. Random effects were systolic blood pressure, diastolic blood pressure, fasting blood glucose, total cholesterol, smoking, alcohol consumption, and CCI score.

**Table 5:** Differences in mean BMI between pre- and 2-year-post-study of TMJD in TMJD II and comparison II group according to obesity.

Characteristics	TMJD II			Comparison II			Interaction ‡	Linear Mixed Model ¶	
	Previous (Mean, SD)	Post 1 Year (Mean, SD)	*p*-Value	Previous (Mean, SD)	Post 1 Year (Mean, SD)	*p*-Value	*p*-Value	EV §	*p*-Value
Underweight (*n* = 190)	17.52 ± 0.88	18.58 ± 1.86	0.003 *	17.50 ± 0.91	18.13 ± 1.72	<0.001 *	0.202	0.467	0.061
Normal (*n* = 3185)	21.39 ± 1.14	21.56 ± 1.51	<0.001 *	21.38 ± 1.14	21.63 ± 1.68	<0.001 *	0.151	−0.071	0.248
Overweight (*n* = 2360)	23.96 ± 0.57	23.90 ± 1.59	0.355	23.98 ± 0.57	23.94 ± 1.40	0.292	0.574	−0.039	0.488
Obese I (*n* = 2225)	26.66 ± 1.25	26.24 ± 1.87	<0.001 *	26.69 ± 1.25	26.45 ± 1.88	<0.001 *	0.010 †	−0.200	0.017 †
Obese II (*n* = 145)	33.08 ± 6.27	30.84 ± 4.10	0.139	31.96 ± 3.26	31.33 ± 2.83	0.057	0.095	−0.541	0.460

Abbreviations: CCI, Charlson Comorbidity Index; EV, estimated value; TMJD, temporomandibular joint disorders. * Paired *t*-test; significance was defined as *p* < 0.05/2. † Linear mixed model; significance was defined as *p* < 0.05/2. ‡ Interaction effects between time and group. § Estimated value of the linear mixed model for the TMJD II group based on the comparison II group. ¶ Fixed effects were age, sex, income, region of residence, TMJD, and time of measurement. Random effects were systolic blood pressure, diastolic blood pressure, fasting blood glucose, total cholesterol, smoking, alcohol consumption, and CCI score.

## Data Availability

Releasing of the data by the researcher is not legally permitted. All data are available from the database of the Korea Center for Disease Control and Prevention. The Korea Center for Disease Control and Prevention allows data access, at a certain cost, for any researcher who promises to follow the stipulated code of research ethics. The data of this article can be downloaded from the website after agreeing to follow the code.
